# Lessons Learned From Implementing Digital Health Tools to Address COVID-19 in LMICs

**DOI:** 10.3389/fpubh.2022.859941

**Published:** 2022-04-08

**Authors:** Caitlyn Mason, Siobhan Lazenby, Rachel Stuhldreher, Meredith Kimball, Rebecca Bartlein

**Affiliations:** Gates Ventures LLC, Exemplars in Global Health, Seattle, WA, United States

**Keywords:** digital health, SDGs, mHealth, COVID-19, LMICs

## Abstract

As COVID-19 strained health systems around the world, many countries developed or adapted digital health tools to detect and respond to the novel coronavirus. We identified transferable lessons from an assessment of implementation factors that led to the rapid launch and scale-up of eight digital tools in low- and middle-income countries during the COVID-19 pandemic. These lessons should inform the development of digital health tools to support public health objectives such as the Sustainable Development Goals. Using the mHealth Assessment and Planning for Scale Toolkit, we assessed the implementation of eight digital tools through desk research and stakeholder interviews. Three core lessons emerged from our findings: (1) user-centered design is key to the widespread adoption of digital tools; (2) strong, country-led partnerships are essential for scaling up and sustaining digital tools; and (3) using adaptable digital tools enables implementers to focus on the content of the solution rather than the technology. Lessons learned from implementing and adapting digital tools quickly during the COVID-19 pandemic can inform the use of digital tools for additional health applications, such as bolstering primary health care, reaching vulnerable and marginalized populations, and empowering health workers with the real-time information necessary to optimize their work and improve the health of their target populations. Future efforts should focus on robust monitoring and evaluation of digital tools and sustainable financing models.

## Introduction

Many digital health programs were in place before the COVID-19 pandemic began in 2020, including those designed to respond to disease outbreaks. As COVID-19 challenges health systems around the world, innovators have developed and adapted digital tools for case management, contact tracing, evidence-based surveillance, training, risk communication, and vaccine delivery (1).

Digital health tools facilitate efficiencies and enable rapid scale-up, near-instantaneous data sharing, and quick data aggregation and analysis. If we can sustain and replicate lessons learned from implementing digital tools during the pandemic, we can leverage their potential to ensure healthy lives and promote well-being for all.

We reviewed digital health solutions in lower- and lower-middle-income countries across three user groups—health care providers, health system managers, and health system clients (2)—before selecting eight examples for assessment (see Supplementary Table 1).

**Figure 1:**
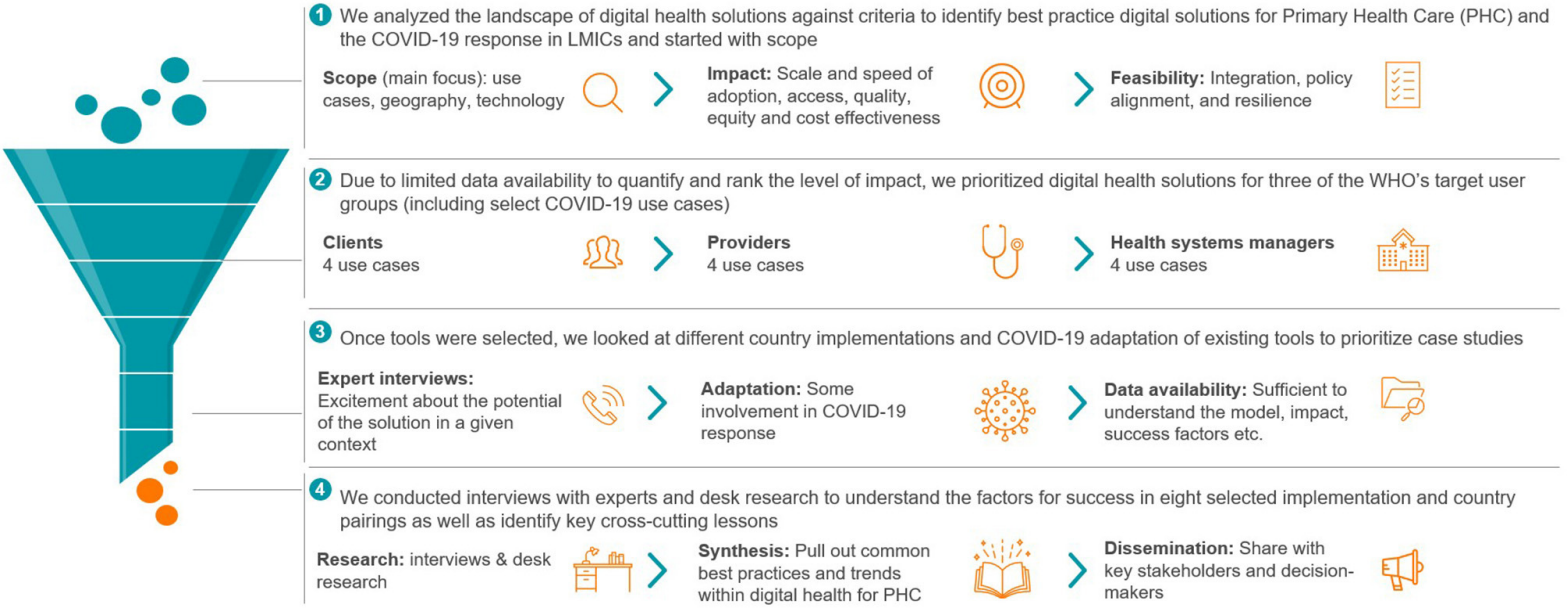
Methodology used to select the digital tools for this assessment.

The selection of eight case studies was driven by scale, impact, and sustainability, as well as availability of information (Figure 1). The case studies draw on desk research and stakeholder interviews with developers, implementers and government representatives, and local experts. The tools we assessed do not necessarily meet all metrics of success, nor do they capture the extent of work done worldwide to implement digital tools as part of the COVID-19 response.

With the right context and committed users, digital tools have the potential to improve health systems, ensure healthy lives, and promote wellbeing; however, most impact-related research has been conducted on smaller projects or specific digital health applications (3). Researchers have found that while some interventions achieve their desired outcomes, many have not been measured. Measuring the impact of digital tools on the pandemic response has been particularly challenging. The need to respond rapidly in a crisis setting limits the ability to evaluate tools' usage. In the absence of true impact measurements, we used alternative metrics as near-term output proxy indicators, including time to deployment, number of laboratories integrated into a system, and number of people reached.

## Framework for Assessment

**Figure 2:**
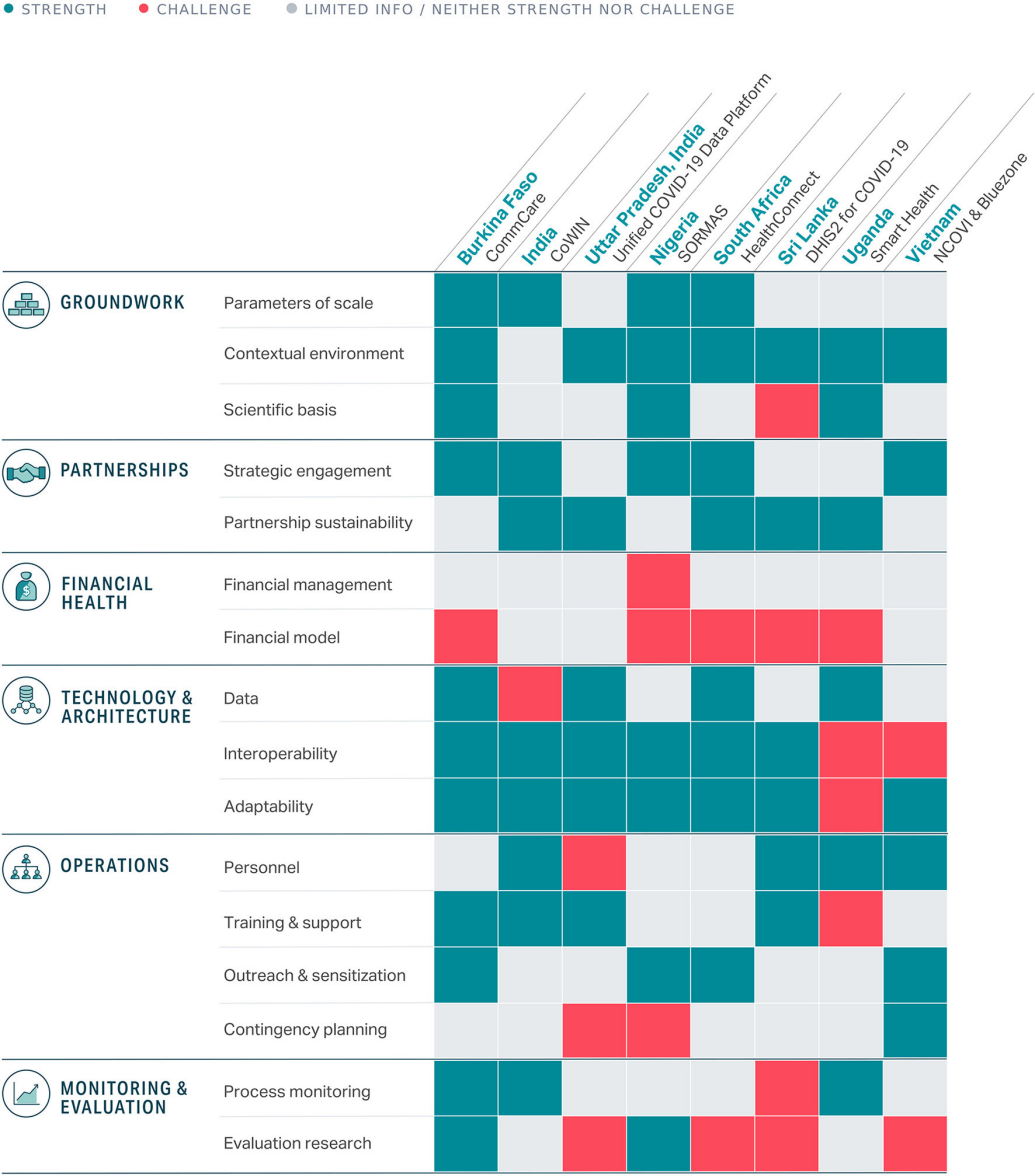
Summary of strengths and challenges across the eight digital tools assessed (detailed descriptions available in Supplementary Table 2).

Multiple frameworks and guides have been developed to support implementation of digital health tools from organizations like the World Health Organization, PATH (Digital Square), and UNICEF (4–6). Of the existing frameworks, we selected the mHealth Assessment and Planning for Scale (MAPS) Toolkit to anchor our analyses because it provided a comprehensive assessment and an actionable guide to scale innovations and maximize their impact. The framework identifies six axes for assessing the implementation and scalability of a given program which we used to assess the performance of digital tools in the sections that follow: groundwork, partnerships, financial health, technology and architecture, operations, and monitoring and evaluation (see Figure 2; Supplementary Table 2) (5).

Our research focused on assessing the implementation of the tools rather than the technology itself. We aimed to supplement the growing knowledge base surrounding best practices in digital health to sustain and strengthen health systems toward Sustainable Development Goal 3: Ensure healthy lives and promote wellbeing for all at all ages (7).

### Groundwork

#### Define the Use Case and Value of the Tool

What is the problem this tool will solve, and for whom? Clear answers to these questions are critical to obtaining buy-in from partners, donors and end-users. All digital tools assessed had a clearly articulated vision.

- In Uttar Pradesh, India, officials worked closely with the Uttar Pradesh Technical Support Unit team to conceptualize and develop the Unified COVID-19 Data Platform—a comprehensive, integrated digital tool for all stakeholders to track and manage the state's COVID-19 response. Within 2 months, the platform evolved into a modular, end-to-end solution for case reporting and management, contact tracing, workflow integration, data aggregation, stakeholder engagement, and strategic planning (8).
- In Uganda, e-learning modules were developed on separate platforms to maintain usability and refresh the knowledge of users (9).

“Using a people-centered design approach, Medic spent several weeks at the community level to understand workflows, how CHWs [community health workers] perform their jobs, potential pain points, communication channels, and infrastructure that would be required to support a digital platform.” (Medic Implementing Partner)

#### Design Human-Centered Solutions Aligned With User Priorities

Successful digital tools effectively address user needs in their design. Pilot testing has ensured that these tools work from a technical perspective and are user-friendly for target audiences.

- Partners in Nigeria worked together to develop the Surveillance Outbreak Response Management and Analysis System (SORMAS). As part of this effort, they addressed challenges related to the country's Ebola response by interviewing stakeholders across all levels of the health system and running multiple pilots throughout the tool's development (10).
- The Vietnamese government worked closely with developers to implement several tools that addressed user feedback. Some of the first location-tracking tools raised concerns about data privacy and mass surveillance, so they developed Bluezone, which supports contact tracing using Bluetooth (11).

#### Understand the Technology's Enabling Environment

Planning for a program's future involves understanding the local context and proactively identifying roadblocks that might hamper progress.

- The Ministry of Health in Sri Lanka launched a master's degree program in health informatics over a decade ago to build a foundation of expertise in using the national health data systems (12). This investment enabled rapid action early in the COVID-19 pandemic.
- After working in South Africa through the MomConnect program for nearly 7 years, the non-profit Praekelt easily navigated local challenges and collaborated with longtime government partners to address challenges through its HealthConnect app (13). The app is available *via* WhatsApp and USSD (a type of text message) for those without data plans, and partnerships with telecommunication providers ensure free access to the messages.

### Partnerships

#### Establish Relationships and Credibility Through Partners, Especially Government

All eight tools we assessed relied on strong government commitment to expand their reach. By working with organizations that already have a presence in-country, developers can draw on their expertise and connections.

- In Burkina Faso's launch of CommCare, the implementing team (led by Terre des hommes) worked closely with the Ministry of Health for more than a decade to create the tool (14). As a result, it has been fully integrated with the country's health infrastructure and transmits data for key indicators automatically to the government's health information system. The tool is considered a national priority in the country's digital health strategy.

“Early on, key people within the MOH believed in the transformative potential of digital health for PHC, and they played a pivotal role in the design, influencing decision makers, and drumming up demand. A chief medical officer involved with the first pilot in Tougan District later transitioned to the central level of the MOH and continues to be an important advisor and ambassador for scaling up the project.” (Implementing partner at Terre des hommes)

- Collaboration between public and private stakeholders drove quick statewide development and adoption of the Unified COVID-19 Data Platform in Uttar Pradesh, India (10). The Department of Health and Family Welfare and the Directorate of Medical Education and Training coordinated the state's COVID-19 response together, and the medium- to long-term thinking and support from technical support partners enabled quick development, implementation, adaptation, and rollout of the digital platform.

### Financial Health

#### Develop Business Models Beyond Donor Investment by Demonstrating Value

Digital tools that charge for services can establish a funding stream from users—either clients or their insurance providers—rather than relying on donors. This applies primarily outside of a crisis such as the COVID-19 pandemic when facilitating quick access is the priority. Of the eight digital tools we assessed, financial health and sustainability was a challenge shared by most.

#### Digitize and Standardize Processes to Establish a Predictable Value

Implementers can demonstrate financial value by lowering costs and better calculating and standardizing the value their products provide to the health system.

- Living Goods and Medic have automated and standardized task and decision support checklists for community health workers in Uganda with the HealthConnect app (9). As part of this effort, they are shifting the workflow from reactive to proactive outreach—in part by using predictive modeling—to improve the impact of the community health worker program. Rather than relying on calls from clients, they can identify people and communities who are more likely to require care, thereby improving the efficiency and cost benefits of the program.
- In Burkina Faso, CommCare is used to simplify complex clinical protocols for health workers. Terre des hommes has worked with partners to leverage artificial intelligence and machine learning to make data processing more efficient, improve measurements, and provide health workers with real-time recommendations based on their performance history, as well as generate smart dashboards and predictive models for epidemiological surveillance. This saves valuable time and enables workers and supervisors to increase their reach.

### Technology and Architecture

#### Develop a Reliable and Adaptable Product

Digital health tools must have the capacity to adapt to shifting priorities. All the tools we assessed had to adapt their technology, with some facing more challenges than others.

- SORMAS in Nigeria was designed specifically to be modular and adjust as new pathogens emerge (11). As a result, a module specific to COVID-19 was ready by January 2020 and integrated seamlessly with the existing technology.
- In India, the phased launch of CoWIN was useful in identifying the technical limitations of the platform and the programmatic limitations of the vaccination drive leading up to the official launch in January 2021 (15). CoWIN's adaptable design enabled the technology team to respond to technical challenges by quickly building key features that allowed users to search pin codes and choose their vaccination centers. By July 2021, officials announced that India would offer the CoWIN platform to the world as an open-source “digital public good.”

#### Ensure Interoperability

For digital tools to integrate with the health ecosystem, they need to work with other data systems and technologies.

- Sri Lanka used DHIS2, an open-source health information system, to manage multiple aspects of the country's COVID-19 response, including port-of-entry tracking and contact tracing (13). Because the underlying DHIS2 platform had existing data infrastructure with known flexibility, it could integrate with other systems like those used by immigration officials. DHIS2's interoperability is one reason for its use in more than 70 lower- and middle-income countries.
- Three design principles—intuitive, modular, and integrated—served as a foundation for the Unified COVID-19 Data Platform in Uttar Pradesh, India (10). This modular, workflow-based system enabled the platform to grow and adapt to meet emerging needs for multiple stakeholder groups. For instance, health workers can refer patients to facilities, officials can leverage a decision-making dashboard to perform dynamic modeling, and citizens can access their COVID-19 test results.

### Operations

#### Invest in Local Expertise

Digital health tools often replace or augment existing workflows. Strengthening local capacity and conducting community outreach helps make this transition to digital tools successful and may increase buy-in for the tools.

- The HigherHealth team in South Africa invested heavily in outreach to campus leadership and students because they recognized that working toward a digital screening and passport system required strong community support (14). The team set up a peer network of thousands of students, which resulted in brand recognition and staying power for the HealthCheck app.
- In Sri Lanka, there was a large focus on local developer expertise. The connection between the Health Information Systems Programme, the DHIS2 core team, and the global community of DHIS2 experts helped local developers get valuable feedback on their work and made it possible for other countries to benefit from their pioneering innovations (13).

#### Invest in Capacity to Use the Solution Locally

The sustainability of a tool depends on local capacity and usability in the country context, which requires additional forms of user support such as language accessibility, training, and user guides.

- The partnership for the development of SORMAS in Nigeria resulted in full government ownership of the tool (11). The program is run by a task force at the Nigeria Centre for Disease Control, with the developers and donors providing technical and financial support. The team runs trainings for multiple user types in several languages and is working toward a cascade training approach, in which one group provides training to another group.
- In Vietnam, the NCOVI and Bluezone apps become more effective as more people download them. Promotional messaging in multiple languages encouraged “challenging the virus with the strength of our community,” and the Ministry of Health and Ministry of Information and Communication encouraged all smartphone users to install Bluezone for themselves and three others: “Protect yourself, protect the community” (12).

### Monitoring and Evaluation

#### Measure the Tool's Impact and Maintain an Adaptable Approach to Implementation

- The Integrated e-Diagnostic Approach in Burkina Faso has undergone multiple independent evaluations that suggest improved health outcomes and quality of care. Estimates show the tool saves between US$830,000 and US$1.7 million per year from reduced training times for community health workers and reduced paper consumption (16).
- Living Goods invested in a randomized controlled trial to evaluate the impact of its SmartHealth app on maternal and child mortality in Uganda. Results in 2014 showed a 27 percent reduction in under-five mortality after 3 years, at an estimated cost of US$68 per life saved (17).

## Discussion

The COVID-19 pandemic has catalyzed innovations in digital health, but scaling and sustaining the innovations remains a challenge. Our assessment builds on existing evidence that has stressed the importance of end-user input, stakeholder engagement, adaptability, interoperability, and alignment with the broader health care ecosystem and policy environment (18). Though the path to scale looked different in every context, three core lessons emerged from our findings:

- *User-centered design is key to the widespread adoption of digital tools*. Each development and implementation team identified an ongoing practical problem and established a clear, user-friendly solution—whether those users were health workers or members of the public.
- *Strong, country-led partnerships are essential for scaling up digital tools successfully*. The solutions that scaled up most successfully during the COVID-19 pandemic benefited from close, long-established partnerships with committed governments to establish and promote the tools.
- *Using adaptable digital tools enables implementers to focus on the content rather than the technology*. Within the global digital health community, there has been a push toward the use and development of global goods (19) or tools that are adaptable and designed to be used in many contexts. This flexibility enables implementers to focus on user-centered design and scale. Many of the digital tools studied were in use for many years before the pandemic, which meant that users already had the required equipment and knowledge to begin leveraging solutions for COVID-19 immediately.

Many of the challenges revealed in our assessment of digital tool implementations are also common to many public health programs globally. Few of the tools assessed have established sustainable financing, business models, or methods to evaluate their impact rigorously. Monitoring and evaluation are not typically prioritized during health emergencies, but the long-term sustainability of these tools remains at risk when funding is not guaranteed and when evidence of improved health outcomes is limited or undocumented. Finally, digital health is not a panacea. If a country faces substantial challenges within its health care system, such as a lack of providers or facilities, digital health tools cannot fully close the gap in quality of or access to care.

These eight case studies complement recent efforts to coordinate implementers around guiding principles, such the WHO's Global Strategy on Digital Health, by offering detailed examples of *how* core lessons have been applied to address common challenges.

## Conclusion

While the COVID-19 pandemic has interrupted and reversed progress made on the Sustainable Development Goals (20), it has also proved to be an important time to demonstrate the value and possibility of digital health tools. These findings are relevant for policy makers, donors, and non-governmental leaders seeking to understand best practices for the implementation of digital tools.

Future efforts in digital health should emphasize rigorous monitoring and evaluation of tool implementation and sustainable financing models. If the lessons from implementing digital tools during the COVID-19 pandemic can be sustained and built upon, digital health tools can realize their potential to increase access to health care, increase the quality of care delivered, complement primary health care systems, and strengthen data for public health decision making (21).

## Data Availability Statement

Publicly available datasets were analyzed in this study. This data can be found here: https://www.exemplars.health/emerging-topics/epidemic-preparedness-and-response/digital-health-tools.

## Author Contributions

CM, SL, and RS conducted desk research and stakeholder interviews. CM, SL, and RB wrote the first draft of the manuscript. All authors contributed to conception and design of the study, revised, read, and approved the submitted version.

## Conflict of Interest

CM, SL, RS, MK, and RB were employed by Gates Ventures LLC.

## Publisher's Note

All claims expressed in this article are solely those of the authors and do not necessarily represent those of their affiliated organizations, or those of the publisher, the editors and the reviewers. Any product that may be evaluated in this article, or claim that may be made by its manufacturer, is not guaranteed or endorsed by the publisher.
