# Supplementary material for: Lessons Learned From Implementing Digital Health Tools to Address COVID-19 in LMICs
Source: Front Public Health. 2022 Apr 8;10:859941. doi: 10.3389/fpubh.2022.859941 (PMC9033038; doi:10.3389/fpubh.2022.859941)
Supplement: Supplementary file 1 [file Table_1.DOCX]

# Supplementary Table 1: Summary of Digital Health Tool Implementations Assessed

| Tool (Country) | Implementation Story | Indicators of Scale | Use Case(s), Primary Funding Source(s), User Group(s) |
| --- | --- | --- | --- |
| CommCare (Burkina Faso) | Scale of an existing app enabled rapid adaptation for COVID-19 | CommCare has scaled to over 1,400 primary care facilities in Burkina Faso and is used by over 6,300 health care workers | Case management  - Donor funding - Health care providers |
| Co-WIN (India) | Leveraging a digital platform for India’s COVID-19 vaccination response | By January 2022, the platform had over 940 million registered users; CoWIN helped administer 1 billion vaccine doses over nine months | Vaccine supply chain, registration and certificationGovernment and donor fundingHealth system managers, health care providers, clients |
| Unified COVID-19 Data Platform (Uttar Pradesh, India) | An integrated end-to-end surveillance platform for COVID-19 response | As of July 2021, the platform enabled the state of Uttar Pradesh to track nearly 49.6 million potential cases across the continuum of care and manage 1.7 million positive cases | Surveillance, contact tracing, facility and lab management  - Government and donor funding  Health system managers, health care providers, clients |
| SORMAS (Nigeria) | Adapting a fully integrated surveillance system to track COVID-19 | SORMAS has been rolled out to all 36 states and the Federal Capital Territory; it has also rolled out in countries including Ghana, Germany, and Fiji | Routine surveillance  - Donor funding, transitioning to country sources - Health system managers |
| HealthConnect (South Africa) | Use of messaging app to enable symptom checking and information across sectors | One of the HealthConnect tools, HealthCheck, has supported over 10 million higher education campus screenings | Risk communication and community engagement  - Donor funding, transitioning to country sources - Clients |
| DHIS2 COVID-19 package (Sri Lanka) | Early action to track and prevent COVID-19 | The Port of Entry module was developed and deployed within a few days of the first reported cluster of COVID-19 cases | Data assets (health management information system)  - Multiple funding sources for COVID-19 modules, including the government and implementer - Health system managers |
| SmartHealth (Uganda) | A mobile app to support community health workers in pandemic response and primary care | Over 7,800 community health workers use the SmartHealth app in Uganda, and referrals and treatments for common conditions (e.g., for childhood pneumonia) increased from 2019 to 2020 | Case management  - Donor funding, experimenting with alternatives - Health care providers |
| NCOVI, Bluezone, others (Vietnam) | Government launches digital health apps to contain COVID-19 | As of March 2021, there were over 30 million downloads of the Bluezone app; by August 2020, NCOVI had over 7.5 million downloads | Contact tracing, risk communication, and community engagementGovernment funding with private sector support  - Clients, health system managers |

# Table 2. Summary of Digital Health Tool Implementations Strengths and Challenges in Six Areas

| **CommCare (Burkina Faso)** | | | | | | | | | | |
| --- | --- | --- | --- | --- | --- | --- | --- | --- | --- | --- |
| Area 1: Groundwork | | | | | | | | | | |
| *Parameters of scale* | | | | | *Contextual environment* | | | | *Scientific basis* | |
| In 2014, the Ministry of Health (MOH) and Terre des hommes (Tdh) leveraged an established digital platform with a proven capacity for scale. By June 2021 the app will be in use at more than 84% of primary health care (PHC) facilities across Burkina Faso. | | | | | The MOH has been involved since the beginning, and the primary implementing partner, Tdh, has a long history in Burkina Faso since 1986. | | | | The app addresses an expressed need to increase adherence to Integrated Management of Childhood Illness (IMCI) protocols and has been used successfully in several other countries. | |
| Area 2: Partnerships | | | | | | | | | | |
| *Strategic engagement* | | | | | | | *Partnership sustainability* | | | |
| Beyond co-creating the Integrated e-Diagnostic Approach (IeDA) with the MOH, Tdh has developed strategic partnerships for operations, research, and funding (for example, with Dimagi, the London School of Hygiene and Tropical Medicine, and the Bill & Melinda Gates Foundation). | | | | | | | From the first pilot, key champions within the government believed in the transformative potential of digital health for PHC. The app is considered a national priority within Burkina Faso’s digital health strategy. | | | |
| Area 3: Financial health | | | | | | | | | | |
| *Financial management* | | | | | | | *Financial model* | | | |
| The London School of Hygiene and Tropical Medicine conducted a cost-effectiveness study in 2016, which showed a 50% reduction in training costs and an estimated annual savings of up to US$1.6 million if scaled up to all regions. | | | | | | | The IeDA project works with an extensive range of partners but is ultimately reliant on donor support. This challenge is likely to persist even after its transition to the MOH. | | | |
| Area 4: Technology and architecture | | | | | | | | | | |
| *Data* | | | *Interoperability* | | | | | | *Adaptability* | |
| Access to data for health workers and government decision makers is critical. Dashboards enable nearly real-time monitoring and targeted supervision, and the app uses artificial intelligence to identify ways to improve worker performance. | | | The app is fully integrated into the country’s health system, with data on key indicators sent automatically to the government’s health information system (DHIS2) for national-level monitoring. It also works with apps for point-of-care diagnosis and safe deliveries. | | | | | | The app’s scope has expanded to include additional modules to address health system needs (e.g., malaria, maternal health, COVID-19), thanks to CommCare’s versatile platform, an iterative approach, and robust user testing. | |
| Area 5: Operations | | | | | | | | | | |
| *Personnel* | *Training and support* | | | | | | *Outreach and sensitization* | | | *Contingency planning* |
| The app generates value for users at every level of the health system. The government, Tdh, and a national committee are dedicated to documenting, staffing, and expanding the app. | CommCare generates timestamps on app usage, enabling targeted supervision, tailored trainings, and e-learning modules to refresh user knowledge. Dimagi strengthened Tdh and MOH capacity to maintain and modify the app. | | | | | | The app facilitates the tasks of overburdened health workers, and most are unwilling to return to paper-based protocols. There also appears to be demand from parents, guardians, and districts outside Tdh zones offering to self-fund the project. | | | The app’s ability to be used offline is instrumental in regions with connectivity challenges. All PHC facilities have solar panels, and a robust troubleshooting hierarchy and regional repair teams address bugs and broken equipment. |
| Area 6: Monitoring and evaluation | | | | | | | | | | |
| *Process monitoring* | | | | | | | *Evaluation* | | | |
| The app gradually scaled up district by district over a decade, introducing new modules via pilots across Burkina Faso. Evidence shows clear impact since the outset of the project. | | | | | | | Multiple independent evaluations have been conducted on health impact, quality of care, and cost savings. CommCare users receive nearly real-time feedback on their performance. The impact in the context of COVID-19 has yet to be evaluated. | | | |
| **CoWIN (India)** | | | | | | | | | | |
| Area 1: Groundwork | | | | | | | | | | |
| *Parameters of scale* | | | | | *Contextual environment* | | | | *Scientific basis* | |
| CoWIN was built with a clear mandate to vaccinate the 1.4 billion population and scale quickly. The platform is an open source, digital public good, designed for widescale adoption by countries around the world. | | | | | Existing digital infrastructure and solutions such as Digilocker, DIVOC, UMANG were leveraged for the development of CoWIN. However, gaps in digital penetration & literacy in India are yet to be bridged. | | | | Pilots were conducted before rolling out the platform for public use. The platform was rolled out in a phased manner for priority beneficiaries, which allowed for iteration and troubleshooting. | |
| Area 2: Partnerships | | | | | | | | | | |
| *Strategic engagement* | | | | | | | *Partnership sustainability* | | | |
| CoWIN is managed by the Ministry of Health and Family Welfare (MoHFW). Partnerships amongst ministries were coordinated by the National Expert Group on Vaccine Administration for COVID-19 (NEGVAC). Experts involved in building other digital public goods in India were also leveraged to implement and scale CoWIN. | | | | | | | The Government of India (GoI), through the National Health Authority (NHA)–with strong support from international partners like the BMGF are in the process of scaling up of the platform. Information on governance of partnerships is limited. | | | |
| Area 3: Financial health | | | | | | | | | | |
| *Financial management* | | | | | | | *Financial model* | | | |
| Though the CoWIN technology is freely available, there is limited information on the total cost of owning and managing CoWIN. Due to the complexities of urgent outbreak response, total expenses have been difficult to define. | | | | | | | Limited information and under development. | | | |
| Area 4: Technology and architecture | | | | | | | | | | |
| *Data* | | | *Interoperability* | | | | | | *Adaptability* | |
| Data generated by CoWIN is stored by Amazon Web Services, accessed via real time dashboard and its quality ensured by disallowing offline data entry. However, data privacy is a concern due to evolving data protection laws in India. | | | CoWIN is interoperable with other apps including Aarogya Setu/UMANG for registration, DIVOC for certification generation, and SAFEVAC for AEFI reporting and with Digilocker for accessing and storing certificates. It is compatible with national databases owned by NHA and MoHFW. | | | | | | CoWIN’s modular design enables the rapid adaptation of content and technology. This has helped India offer CoWIN as a digital global good for other countries to adopt. | |
| Area 5: Operations | | | | | | | | | | |
| *Personnel* | *Training and support* | | | | | | *Outreach and sensitization* | | | *Contingency planning* |
| CoWIN leveraged existing healthcare personnel such as ASHA workers, whose roles & responsibilities were delineated by NEGVAC. Each district also had a single point of contact. | Staff working on COVID-19 vaccination were provided with online and onsite training. User Helplines for troubleshooting were also introduced for users & staff members working on implementation of the vaccination drive. | | | | | | Stakeholder outreach and community mobilization was carried out by GoI, international partners, and other nongovernmental bodies. | | | As per operational guidelines, there are provisions for contingency plans but information is limited. |
| Area 6: Monitoring and evaluation | | | | | | | | | | |
| *Process monitoring* | | | | | | | *Evaluation* | | | |
| Performance monitoring of CoWIN is conducted daily using established monitoring formats as per the operational guidelines for COVID-19 management. The workflows for management of administrative staff are clearly defined and data entry is monitored at the backend to minimize errors. | | | | | | | No evaluation of CoWIN has been conducted as of January 2022. | | | |
| **Unified COVID-19 Data Platform (Uttar Pradesh, India)** | | | | | | | | | | |
| Area 1: Groundwork | | | | | | | | | | |
| *Parameters of scale* | | | | | *Contextual environment* | | | | *Scientific basis* | |
| Initially, developers chose a local tech vendor for development before realizing that this wouldn’t achieve the desired scope and scale. | | | | | The developers had a strong understanding of the available resources, user workflows, and the continuum of care across departments. | | | | Early on in the development, officials included stakeholders (e.g., laboratory, surveillance and facility teams) in the design process to ensure the system met their needs and added value. | |
| Area 2: Partnerships | | | | | | | | | | |
| *Strategic engagement* | | | | | | | | *Partnership sustainability* | | |
| There was strong and coordinated support from state government leaders from the Department of Health and Department of Medical Education under the guidance of the Honorable Chief Minister’s office. | | | | | | | | Collaboration between the government and partner organizations drove quick statewide development and adoption of the platform. | | |
| Area 3: Financial health | | | | | | | | | | |
| *Financial management* | | | | | | | | *Financial model* | | |
| There was a memorandum of understanding between the government of Uttar Pradesh and the Bill & Melinda Gates Foundation. | | | | | | | | Limited information and under development. | | |
| Area 4: Technology and architecture | | | | | | | | | | |
| *Data* | | | | *Interoperability* | | | | | *Adaptability* | |
| The ability to view and analyze case trends in real time is an important contribution of the platform. Policymakers are equipped to make data-driven decisions (e.g., increasing beds). | | | | The platform integrates central/state platforms and provides an end-to-end solution that became the single source of truth across the state for COVID-19 management. | | | | | The platform’s modular design integrates the needs of various stakeholders, from tracking teams to testing labs to patients in quarantine. This design enabled the platform to adapt to a variety of use cases, emerging health priorities, and system needs. | |
| Area 5: Operations | | | | | | | | | | |
| *Personnel* | | *Training and support* | | | | | | *Outreach and sensitization* | | *Contingency planning* |
| Sustaining capacity of a digital team to lead, manage, and support the development of systems and platforms has been a challenge. | | Remote trainings for health care workers can be adapted for future disease outbreaks and community health workers. Videos were posted to YouTube and WhatsApp. | | | | | | Uptake enhanced by establishing a user-friendly, comprehensive service delivery and reporting process. There is a need for more direct-to-citizen communication to drive user engagement. | | Initially, the platform faced system performance issues. Plans to handle broken devices and offline requirements are evolving. |
| Area 6: Monitoring and evaluation | | | | | | | | | | |
| *Process monitoring* | | | | | | | | *Evaluation* | | |
| The platform’s built-in validation and data quality checks have been crucial for transparency and continuous improvement. | | | | | | | | No impact evaluation or cost-effectiveness studies have been conducted as of July 2021. | | |
| **SORMAS (Nigeria)** | | | | | | | | | | |
| Area 1: Groundwork | | | | | | | | | | |
| *Parameters of scale* | | | | | *Contextual environment* | | | | *Scientific basis* | |
| SORMAS had a clear purpose from the outset: to address challenges uncovered during the 2014 Ebola outbreak. Use of the tool expanded with further outbreaks, in collaboration with partners in real time. | | | | | Throughout the design process, many different end users were interviewed to understand tools that had previously been used. | | | | Multiple rounds of user testing were conducted during the pilot phase and the design process brought in a variety of local perspectives across government, academia, and other entities. | |
| Area 2: Partnerships | | | | | | | | | | |
| *Strategic engagement* | | | | | | | | *Partnership sustainability* | | |
| SORMAS was co-created with local stakeholders, including the Nigeria Centre for Disease Control (NCDC) and the African Field Epidemiology Network (AFENET) to ensure joint ownership. One challenge is obtaining support from other government agencies to ensure full data sharing. | | | | | | | | The Helmholtz Centre for Infection Research (HZI) and German Corporation for International Cooperation (GIZ) support the continued scale-up of the tool. Information on governance procedures is limited, but major decisions are led by the NCDC, with strong support from within the organization. | | |
| Area 3: Financial health | | | | | | | | | | |
| *Financial management* | | | | | | | | *Financial model* | | |
| The cost of running SORMAS is unclear and difficult to budget for. This is driven in part by the complexities of managing administrative needs with quick outbreak response. | | | | | | | | SORMAS in Nigeria depends on donor funding, particularly from GIZ, which could pose challenges to long-term stability if donor priorities shift. | | |
| Area 4: Technology and architecture | | | | | | | | | | |
| *Data* | | | | *Interoperability* | | | | | *Adaptability* | |
| A nationally owned data store is mandated. Early on, SORMAS lacked high-quality data protections, but this is shifting with the implementation of SORMAS in Europe, driving increased data security for all implementations. | | | | SORMAS interacts both with systems at the country level (e.g., District Health Information Software 2 [DHIS2] and Logistics Management Systems [LMIS]) and allows for easier data sharing at the regional level (e.g., West African Health Organization). It is compatible with other country epidemiological data systems like the electronic Integrated Disease Surveillance and Response system. | | | | | SORMAS has demonstrated the ability to quickly include new disease modules as needed (e.g., the COVID-19 module was available in January 2020) and to function across different devices. | |
| Area 5: Operations | | | | | | | | | | |
| *Personnel* | | *Training and support* | | | | | | *Outreach and sensitization* | | *Contingency planning* |
| The NCDC primarily runs SORMAS, with support from HZI. In times of major outbreaks, gaps in staffing lead to challenges in managing national-level surveillance. | | Online training modules are available for users, and in-person training is typically provided. Some challenges exist in reaching people during the COVID-19 pandemic and getting full participation. | | | | | | The development team engaged with different groups throughout the development process. | | The app can be used offline on mobile devices, but there is limited information on broad engagement with the private sector to mitigate costs of data and internet access. |
| Area 6: Monitoring and evaluation | | | | | | | | | | |
| *Process monitoring* | | | | | | | | *Evaluation* | | |
| Workflows are clearly defined and data inputs are evaluated. Internal efforts have attempted to assess and improve data collection, but evaluations have been deprioritized due to the COVID-19 pandemic response. | | | | | | | | Multiple evaluations were conducted before the COVID-19 pandemic. HZI and GIZ have documented and shared efforts widely. Ongoing efforts intend to expand operational research. | | |
| **HealthConnect (South Africa)** | | | | | | | | | | |
| Area 1: Groundwork | | | | | | | | | | |
| *Parameters of scale* | | | | | *Contextual environment* | | | | *Scientific basis* | |
| The goal of HealthConnect was to build an information platform for South Africa’s citizens. Over time, the parameters changed to adapt to the changing state of the COVID-19 pandemic. | | | | | Given the strong partnership already in place, implementers and government stakeholders were well-versed in South Africa’s digital ecosystem. | | | | The impact of on-demand information can be measured by uptake. However, the value of other features (e.g., HealthCheck) is unknown. | |
| Area 2: Partnerships | | | | | | | | | | |
| *Strategic engagement* | | | | | | *Partnership sustainability* | | | | |
| An existing partnership between Praekelt and the National Department of Health (NDOH) enabled quick engagement to develop a tool for COVID-19 in March 2020. Early success led to the new engagements with other government agencies (e.g., HigherHealth). | | | | | | Praekelt and the NDOH have a relationship dating back to 2014, when they co-developed MomConnect. COVIDConnect has expanded this collaboration. | | | | |
| Area 3: Financial health | | | | | | | | | | |
| *Financial management* | | | | | | *Financial model* | | | | |
| The costs of running HealthConnect (and similar platforms) is well understood at approximately US$1-2 per user. However, the cost per user is highly dependent on the total user base, and the program requires significant scale to be cost-effective. | | | | | | Donor funding and cost breaks from telecom providers were crucial to the implementation of HealthConnect and before that, MomConnect. Long-term sustainability of the tool will depend on the ability to secure a stable funding source. | | | | |
| Area 4: Technology and architecture | | | | | | | | | | |
| *Data* | | | | *Interoperability* | | | | | *Adaptability* | |
| Data from all tools rolls into national databases owned by the NDOH and National Institute for Communicable Diseases (NICD). This data is then aggregated in dashboards that can be used for decision making at multiple levels of government. | | | | To assist with contact tracing, HealthConnect connects with the main NDOH and NICD databases and the laboratory information systems. | | | | | The underlying technology (turn.io) is easily adapted and updated based on the latest information and specific needs. As a result, the HealthConnect suite of tools can be used across sectors. | |
| Area 5: Operations | | | | | | | | | | |
| *Personnel* | | *Training and support* | | | | *Outreach and sensitization* | | | | *Contingency planning* |
| Limited information is available. The NDOH central team is familiar with MomConnect. | | The direct-to-consumer nature of the tool (in most cases) requires minimal training. | | | | HealthConnect relies on heavy community outreach. Early on, the phone number was printed on bread packaging and ran on news tickers. HigherHealth did several radio shows to reach users through familiar channels. | | | | The tool has limited applicability. It does not require new equipment. HealthConnect’s platform is designed to be adaptable on the back end for changing needs. |
| Area 6: Monitoring and evaluation | | | | | | | | | | |
| *Process monitoring* | | | | | | *Evaluation* | | | | |
| Limited information is available on how uptake is monitored beyond access data or what drives continued use of the tool. | | | | | | Research on the impact of HealthConnect has not been conducted to date, and neither Praekelt nor the government agencies have outlined public plans to evaluate the data collected. | | | | |
| **DHIS2 for COVID-19 (Sri Lanka)** | | | | | | | | | | |
| Area 1: Groundwork | | | | | | | | | | |
| *Parameters of scale* | | | | | *Contextual environment* | | | | *Scientific basis* | |
| The scope of need was unclear because the work began before COVID-19 was well understood. | | | | | The Sri Lanka MOH has been investing in health information expertise for years. The strong Health Information Systems Programme (HISP) hub in Sri Lanka enabled effective, rapid action. | | | | Due to the short timeline, there was no opportunity for the desired pilot period, but the team received input from select close partners before scaling up nationally. | |
| Area 2: Partnerships | | | | | | | | | | |
| *Strategic engagement* | | | | | | *Partnership sustainability* | | | | |
| The project was commissioned by the MOH and executed by HISP in Sri Lanka, with support from the Global HISP network. | | | | | | An HISP team based in Sri Lanka supports continued scale-up of the tool. The team has a strong relationship with the MOH and was the clear partner for co-developing a digital COVID-19 response. | | | | |
| Area 3: Financial health | | | | | | | | | | |
| *Financial management* | | | | | | *Financial model* | | | | |
| No clear funding has been allocated to developing the COVID-19 modules. The team developed the new modules cost-effectively, in part through a “hackathon” of volunteer contributors. | | | | | | Limited information is available. The long-term financial model is unclear. | | | | |
| Area 4: Technology and architecture | | | | | | | | | | |
| *Data* | | | | *Interoperability* | | | | | *Adaptability* | |
| The MOH used the data collected to inform decisions throughout 2020. | | | | Each module was built to be interoperable with DHIS2 and other key information systems (such as immigration), which enabled quick training and uptake. | | | | | The DHIS2 platform is easily adaptable, enabling rapid development and adjustment to modules. Because the scope of requirements was not immediately clear, the adaptability of the system was a key driver in building on DHIS2. | |
| Area 5: Operations | | | | | | | | | | |
| *Personnel* | | *Training and support* | | | | *Outreach and sensitization* | | | | *Contingency planning* |
| Having a team on the ground in Sri Lanka with a strong connection to the MOH was a critical enabler of the strong response. | | The HISP team rapidly developed and deployed trainings for users. | | | | Due to the speed of deployment, there was not enough time to educate users before incorporating the modules into existing processes. | | | | Limited information is available. The team reacted quickly to changing pandemic requirements. |
| Area 6: Monitoring and evaluation | | | | | | | | | | |
| *Process monitoring* | | | | | | *Evaluation* | | | | |
| While the team provides active technical support, the need to move quickly on new modules was prioritized over monitoring existing modules. | | | | | | Limited information is available on efforts to evaluate the tool’s impact during the COVID-19 pandemic. | | | | |
| **Smart Health (Uganda)** | | | | | | | | | | |
| Area 1: Groundwork | | | | | | | | | | |
| *Parameters of scale* | | | | | *Contextual environment* | | | | *Scientific basis* | |
| Medic partnered with Living Goods to scale the Smart Health app, based on the existing Community Health Toolkit for all their community health workers. | | | | | Living Goods was familiar with the community health worker workflows and Ugandan context; it began operations in the country seven years before partnering with Medic. The Development team spent weeks on the ground to better understand Living Good’s challenges. | | | | Living Goods invested in randomized control trials to prove the efficacy of the app, finding reductions in costs and child mortality. | |
| Area 2: Partnerships | | | | | | | | | | |
| *Strategic engagement* | | | | | | *Partnership sustainability* | | | | |
| Living Goods engages closely with the Ministry of Health and other health NGOs in Uganda (e.g., BRAC). | | | | | | The pandemic has provided a unique opportunity for Living Goods to strengthen its partnership with the MOH. It has seconded staff to the ministry and to call centers, donated personal protective equipment, and created a new partnership to support government community health workers. | | | | |
| Area 3: Financial health | | | | | | | | | | |
| *Financial management* | | | | | | *Financial model* | | | | |
| The financial model is based on consulting, through subcontracts with traditional funders for implementation. | | | | | | Financing is currently unsustainable; Living Goods still relies on donor contributions to maintain operations, despite new innovative co-financing contracts with governments. Full financial sustainability will require massive scale-up of these service-type contracts. | | | | |
| Area 4: Technology and architecture | | | | | | | | | | |
| *Data* | | | | *Interoperability* | | | | | *Adaptability* | |
| The app focuses on human, non-technical factors first. Solutions need to have a strong, clearly articulated value for end users, while demonstrably improving quality of care. | | | | The underlying Medic technology is designed to be interoperable; however, integration with DHIS2 in Uganda has faced challenges. | | | | | During the COVID-19 pandemic, the difficulty of translating government COVID protocols into new workflows in some counties led to delays in program adjustments, in some cases not rolled out until June 2020. | |
| Area 5: Operations | | | | | | | | | | |
| *Personnel* | | *Training and support* | | | | *Outreach and sensitization* | | | | *Contingency planning* |
| Supports 7,800 community health workers. Living Goods has dedicated team members to adapt the Smart Health app. | | Training represents Living Goods’ biggest bottleneck to scale, community health workers must be licensed by the government as well as trained to use the platform | | | | Limited information is available. Part of community health worker training. | | | | Offline capabilities are in place to account for limited connectivity. |
| Area 6: Monitoring and evaluation | | | | | | | | | | |
| *Process monitoring* | | | | | | *Evaluation* | | | | |
| Living Goods operations are data driven—the supervisors can monitor community health worker performance, and the app helps prioritize their activities. | | | | | | Living Goods has had multiple randomized control trials performed on its interventions in the past, including the Smart Health app. During COVID-19, they have tracked referrals and treatments provided. | | | | |
| **NCOVI and Bluezone (Vietnam)** | | | | | | | | | | |
| Area 1: Groundwork | | | | | | | | | | |
| *Parameters of scale* | | | | | *Contextual environment* | | | | *Scientific basis* | |
| The MOH and Ministry of Information and Communications (MIC) encouraged immediate use by all citizens and visitors to Vietnam with smartphones. The long-term use of these tools has not been determined. | | | | | The government and developers leveraged lessons learned from SARS. Vietnam also has a commitment to digital transformation, a growing technology sector, and high mobile penetration. Implementation was led by the MOH and MIC. | | | | The more the apps are used, the more effective they are. For NCOVI, there are challenges around the stigma of self-reporting and voluntary symptom logging of asymptomatic transmission. | |
| Area 2: Partnerships | | | | | | | | | | |
| *Strategic engagement* | | | | | | *Partnership sustainability* | | | | |
| Applications were designed at the request of Deputy Prime Minister Vu Duc Dam. Partnerships were between the MOH, MIC, and telecom groups (i.e., Viettel, MobiFone, VNPT, VinaPhone), and technology companies (i.e., Bkav, Memozone). State-owned groups and high levels of government trust influenced speed of development and uptake. | | | | | | Support from key champions in the government has played an integral role in the speed of the tool’s development and scale. The long-term plan is unknown. | | | | |
| Area 3: Financial health | | | | | | | | | | |
| *Financial management* | | | | | | *Financial model* | | | | |
| More information is required on operating costs and total cost of ownership for health system and users. | | | | | | The three largest state-owned telecom groups supported advertising for the apps and free data for subscribers when using the apps. The long-term plan is unknown. | | | | |
| Area 4: Technology and architecture | | | | | | | | | | |
| *Data* | | | | *Interoperability* | | | | | *Adaptability* | |
| Privacy is a concern for the NCOVI app, which prompted development of Bluezone (which does not use location data). Data are leveraged to create hotspot maps to inform users and decision makers. | | | | Lack of interoperability between the many apps being used for surveillance posed a challenge and barrier to scale-up. | | | | | Simple web apps were developed before NCOVI for mobile use. An expanded suite of tools was created to handle new use cases and health system needs, and address limitations. Bluezone is open-source and encourages other countries to use, research, and modify the code. | |
| Area 5: Operations | | | | | | | | | | |
| *Personnel* | | *Training and support* | | | | *Outreach and sensitization* | | | | *Contingency planning* |
| MIC mobilized experts from different technology groups to independently develop and share technology challenges and discuss solutions before selection. | | Given the client-facing nature of the tool, no training is required or provided. A highly connected and tech-savvy population enabled scale-up. | | | | The MOH leveraged Zalo and telecom groups to encourage downloads. Campaigns encouraged users to install themselves and for others: “Protect yourself, protect your community.” | | | | Suite of tools designed to address limitations in previous builds. |
| Area 6: Monitoring and evaluation | | | | | | | | | | |
| *Process monitoring* | | | | | | *Evaluation* | | | | |
| Additional details are required to understand use of the apps beyond the number of downloads (e.g., forms submitted, time on app, reporting for household members). | | | | | | Both NCOVI and Bluezone were used to identify contacts, but their impact (and intentions to evaluate the impact) are unclear. | | | | |

Note: To assess the performance of the eight digital health tools in our assessment, we used a framework from the World Health Organization’s [*MAPS Toolkit: mHealth Assessment and Planning for Scale*](https://apps.who.int/iris/bitstream/handle/10665/185238/9789241509510_eng.pdf),^[[1]](#footnote-1)^ which identifies six axes for assessing the implementation and scalability of a given program: groundwork, partnerships, financial health, technology and architecture, operations, and monitoring and evaluation.

1. World Health Organization (WHO), United Nations Foundation, UNDP/UNFPA/WHO/World Bank Special Programme of Research, Development and Research Training in Human Reproduction & Johns Hopkins University. *The MAPS Toolkit: mHealth Assessment and Planning for Scale*. Geneva: WHO; 2015. <https://apps.who.int/iris/handle/10665/185238> [↑](#footnote-ref-1)
